# Parameter and model uncertainty in a life-table model for fine particles (PM_2.5_): a statistical modeling study

**DOI:** 10.1186/1476-069X-6-24

**Published:** 2007-08-23

**Authors:** Marko Tainio, Jouni T Tuomisto, Otto Hänninen, Juhani Ruuskanen, Matti J Jantunen, Juha Pekkanen

**Affiliations:** 1 Centre of Excellence for Environmental Health Risk Analysis, National Public Health Institute, Kuopio, Finland; 2 Department of Environmental Science, University of Kuopio, Kuopio, Finland; 3 School of Public Health and Clinical Nutrition, University of Kuopio, Kuopio, Finland

## Abstract

**Background:**

The estimation of health impacts involves often uncertain input variables and assumptions which have to be incorporated into the model structure. These uncertainties may have significant effects on the results obtained with model, and, thus, on decision making. Fine particles (PM_2.5_) are believed to cause major health impacts, and, consequently, uncertainties in their health impact assessment have clear relevance to policy-making. We studied the effects of various uncertain input variables by building a life-table model for fine particles.

**Methods:**

Life-expectancy of the Helsinki metropolitan area population and the change in life-expectancy due to fine particle exposures were predicted using a life-table model. A number of parameter and model uncertainties were estimated. Sensitivity analysis for input variables was performed by calculating rank-order correlations between input and output variables. The studied model uncertainties were (i) plausibility of mortality outcomes and (ii) lag, and parameter uncertainties (iii) exposure-response coefficients for different mortality outcomes, and (iv) exposure estimates for different age groups. The monetary value of the years-of-life-lost and the relative importance of the uncertainties related to monetary valuation were predicted to compare the relative importance of the monetary valuation on the health effect uncertainties.

**Results:**

The magnitude of the health effects costs depended mostly on discount rate, exposure-response coefficient, and plausibility of the cardiopulmonary mortality. Other mortality outcomes (lung cancer, other non-accidental and infant mortality) and lag had only minor impact on the output. The results highlight the importance of the uncertainties associated with cardiopulmonary mortality in the fine particle impact assessment when compared with other uncertainties.

**Conclusion:**

When estimating life-expectancy, the estimates used for cardiopulmonary exposure-response coefficient, discount rate, and plausibility require careful assessment, while complicated lag estimates can be omitted without this having any major effect on the results.

## Background

The estimation of health effects of environmental stressors always involves uncertainty. The input variables (data) or the mathematical formulation of the model contain uncertainty, and some information may be missing. The sources of uncertainty can be categorized into parameter and model uncertainties [e.g. [1,2]]. Depending on the situation, uncertainties may have large impacts on model results and, thus, lead to a situation where uncertainties hamper decision-making.

Fine particles (PM_2.5_) have been shown to damage the health. The Clean Air for Europe (CAFE) program, funded by the European Commission, claimed that fine particles cause over 300000 premature deaths annually in Europe and lower the average life-expectancy by 8.6 months [3]. In this respect, air pollution by fine particles is one of the most important environmental health problems in Europe.

The health effects of PM_2.5 _have been assessed using both additional mortality and life-expectancy methods. The additional mortality (estimating the number of premature deaths) method has been used in a number of health risk studies to measure changes in annual or daily mortality [e.g. [4,5]]. Another approach, estimation of the life-expectancy of a population by using a life-table method [6], has also been used [e.g. [7-9]]. The advantage of life-expectancy predictions is that the method predicts correctly the change in the population age structure. The practical downside is that life-expectancy method requires more laborious intensive models and more input variables.

The sensitivity of life-table models to uncertainties in some input variables has been investigated in several studies. Although several models with different assumptions have been created, there is no comprehensive sensitivity analysis of all key assumptions and input variables in the PM_2.5 _life-table models. Brunekreef [7] concluded that the life-expectancy predictions are sensitive to extrapolation of cohort studies results to the older age groups. Nevalainen and Pekkanen [9] compared the loss of life-expectancy due to lung cancer and cardiopulmonary mortality using two different cohort study estimates [10,11]. Their results indicated that the predictions of the health effects differ largely between the different causes and between the estimates obtained from different studies. Rabl claimed that the infant mortality had only a minor effect on life-expectancy [12]. Uncertainty due to lag has been noted to be small when compared with the uncertainties encountered in epidemiological studies [13]. In the present article, lag was defined as the time elapsing between a change in exposure and the ensuing change in the hazard rate. Discounting has been used to express future benefits as present values. It has had varying effects on the results, depending on the outcome being assessed [13,14]. The uncertainty analysis, which was done in CAFE program, contained also input variables from the life-table model (e.g. concentration-response functions, monetary valuation) [15].

In this article, we compare uncertainties of fine particle life-table model attributable to different sources: different health outcomes, lag of the health effects, and change in the exposure. In addition, we used the monetary value of years-of-life-lost and the relative importance of the uncertainties related to monetary valuation (discount and valuation of a life-year-lost). The model was implemented for the year 2002 Helsinki metropolitan area population. The aims of this study were (i) to quantify the effect of the uncertainty on life-table model results and (ii) to compare the relative importance of the different input variables (parameter uncertainty) and model assumptions (model uncertainty).

## Methods

### Overview of the model

The effect of uncertainty was studied by conducting a comprehensive sensitivity analysis of a life-table model. Both parameter and model uncertainties were propagated through the model by Monte Carlo simulation. The model uncertainty was described with binary variables (Bernoulli distribution), choosing between two alternative model branches (exception discount rate). Parameter uncertainty was described by using continuous distributions. The effects of parameter and model uncertainties on model results were studied using sensitivity analysis. The sensitivity analysis was done by calculating absolute rank-order correlations between the input variables and the model results. All the studied input variables are presented in table 1.

**Table 1:** Input variables included in the sensitivity analysis.

Variable	Type of uncertainty	Distribution	Parameters	Explanation and references
Exposure-response coefficient for cardiopulmonary mortality, adults (*RR*)	Parameter uncertainty	Mixed^a^	1.12 (1.04;1.27)	Relative increase of mortality per 10 μgm^-3 ^increase of PM_2.5 _exposure. Values were drawn with equal probability from the two distributions reported in the references Dockery et al. 1993 [10] and Pope et al. 2002 (table 3, average between 1979 and 1999-200 results) [25].
Exposure-response coefficient for lung cancer mortality, adults (*RR*)	Parameter uncertainty	Mixed	1.15 (0.94;1.40)	
Exposure-response coefficient for all other non-accidental mortality, adults (*RR*)	Parameter uncertainty	Mixed	1.01 (0.91;1.09)	
Plausibility, cardiopulmonary mortality	Model uncertainty	Bernoulli^b^	P = 0.7 yes, P = 0.3 no	AJ^c^. Plausibility = "Is the observed effect due to true causal connection."
Plausibility, lung cancer mortality	Model uncertainty	Bernoulli	P = 0.9 yes, P = 0.1 no	
Plausibility, other mortality	Model uncertainty	Bernoulli	P = 0.1 yes, P = 0.9 no	
Exposure-response coefficient for non-accidental mortality, infants (*RR*)	Parameter uncertainty	Normal^d^	1.04; 0.013	Relative increase of infant mortality (less than one year old infants) per 10 μgm^-3 ^increase of PM_2.5 _exposure. Reference Woodruff et al. 1997 [30].
Plausibility, non-accidental mortality, infant	Model uncertainty	Bernoulli	P = 0.6 yes, P = 0.4 no	AJ. Plausibility = "Is the observed effect due to true causal connection."
Lag vs. zero-lag	Model uncertainty	Bernoulli	P = 0.5 yes, P = 0.5 no	AJ. Lag = the time difference between the exposure and the causal health effect. See The lag sub-model chapter for details.
Exposure (μgm^-3^), infants (2002) (Δ*E*)	Parameter uncertainty	Log-normal^e^	0.31; 3.27	The exposure of study population for local traffic related primary fine particles (0–6, 7–59 and 59–110 years for infants, adults and elderly, respectively). The exposure model [19–21] was based on EXPOLIS-Helsinki study [22–24]. See Exposure scenarios sub-model chapter for details.
Exposure (μgm^-3^), adults (2002) (Δ*E*)	Parameter uncertainty	Log-normal	0.56; 2.81	
Exposure (μgm^-3^), elderly (2002) (Δ*E*)	Parameter uncertainty	Log-normal	0.55; 3.08	
Exposure (μgm^-3^), infants (2025) (Δ*E*)	Parameter uncertainty	Log-normal	0.14; 3.56	
Exposure (μgm^-3^), adults (2025) (Δ*E*)	Parameter uncertainty	Log-normal	0.21; 2.94	
Exposure (μgm^-3^), elderly (2025) (Δ*E*)	Parameter uncertainty	Log-normal	0.2; 3.24	
Valuation of a life-year-lost	Parameter uncertainty	Uniform^f^	52000; 120000	Min and max from CAFE study [39]. See text for details.
Discount	Model uncertainty	Uniform	0.02; 0.06	Min and max from CAFE study [39]. See text for details.
Blank	-	Uniform	0.0; 1.0	Blank = internal standard of the model. Not related to the model results.

a Mixed: Combination of two normally distributed variables (mean and 90% confidence intervals).

b Bernoulli (binomial) binary probability distribution with probabilities (P, 1-P)

c AJ = Author judgment.

d Parameters for normal distribution (mean; standard deviation)

e Parameters for log-normal distribution (median; geometric standard deviation)

f Parameters for uniform distribution (min; max)

Life-expectancy (LE) and monetary value of lost life years were predicted. The LE was predicted by defining the change in the background hazard rate caused by local-traffic-related primary fine particles. The effects of fine particles were predicted for both infants and adults. The time difference between an exposure and the consequent health effects (lag) was included in the model. The relative risk and lag estimates are described below. The monetary value of life-year-lost was predicted by calculating a value for a life year and discounting the future benefits and costs. The model was implemented using Analytica ™ version 3.1.1. (Lumina Decision Systems, Inc., CA) Monte Carlo simulation program and run with 5 000 iterations.

### Exposure scenarios sub-model (ΔE_k_)

Exposure scenarios were based on the local traffic-related exposure variables that were defined within the project *Health Effects caused by Urban Air Pollution for the Transport System Plan Scenarios in Helsinki Area *(HEAT). The HEAT project evaluated traffic flows, air pollution emissions from traffic, resulting ambient air concentrations and exposures, and health effects in the Helsinki metropolitan area. The evaluations were done for two years, 2002 (current) and 2025 (the target year of the current transport system plan of this area). The traffic volumes for 2025 were assessed by combining Helsinki metropolitan area land use scenarios with a traffic demand model prepared by the Helsinki Metropolitan Area Council (YTV). The spatial distribution and the amount of key air pollution emissions, including fine particles, were predicted from the traffic flow estimations.

The hourly fine particle concentrations from primary traffic emissions over the Helsinki metropolitan area were computed by Finnish Meteorological Institute [16] within spatial resolution ranging from 10 to 500 meters. Ambient concentrations from local traffic were sampled for residential and occupational locations using a stratified random sampling scheme and small-area planning districts. Indoor concentrations of local traffic related fine particles were modelled using an infiltration model [17]. Concentrations experienced while in traffic were predicted from *EXPOLIS *data [18] and scaled for the years 2002 and 2025 using predicted changes in tailpipe emissions. Population exposures were estimated separately for infants, adults, and elderly (0–1, 25–59, and 60–110 years, respectively). The exposure model [19-21] was based on *EXPOLIS*-Helsinki study [22-24] that measured exposure of adult population to various air pollutants in the study area. Exposures of the infants and the elderly were obtained by combining time activity data with the measured concentration data in relevant microenvironments. In the life-table model, the infant exposure was extended for years 0–6 (below school age) and adult exposure for the years 7–59 (table 1).

The exposure distributions were designed to represent individual variations in the personal exposures. These distributions are likely to be wider than the uncertainty distributions for the age group averages, and therefore they provide an upper limit estimate of the importance of exposure uncertainty.

Four exposure scenarios (i-iv) were defined. (i) 'Current with traffic' scenario assumed that the exposure to local traffic related fine particles would remain at the year 2002 level. This is the base scenario used, and exposure uncertainties were not included for this scenario. This is because the combined effect of fine particle exposure and all other causes of mortality are seen in the current mortality statistics with very high precision and because the main aim is to focus on uncertainties related to fine particles. Therefore, the uncertainties in the current exposure to fine particle are propagated into scenarios ii and iii, which are expressed in comparison to the baseline scenario.

(ii) 'Reduced traffic emissions' scenario assumed that the exposure to fine particles due to local traffic would decline linearly between the years 2002 and 2025 and would remain constant thereafter. (iii) 'Current without traffic' scenario assumed that the exposure to all local-traffic-related fine particles was zero. Thus, the 'Current without traffic' scenario can be used to predict loss of life-expectancy due to traffic-related primary fine particles. (iv) The 'Additional exposure' scenario assumed a constant 10 μgm^-3 ^exposure above the actual 2002 level after the year 2002 to the total population. The 'additional exposure' scenario was used to compare the magnitude of the health effects to those obtained in the other studies. The exposure scenarios and their properties are summarized in table 2.

**Table 2:** The description of different scenarios. See Exposure scenarios sub-model -chapter for details.

	**Scenario**			
	**Current with traffic**	**Reduced traffic emissions**	**Current without traffic**	**Additional exposure**

Follow-up period	Years 2002–2112			
Population	Year 2002 Helsinki Metropolitan Area population			
Primary PM_2.5 _health effects	Included in background mortality	Included in background mortality	Background mortality minus health effects of traffic related primary PM_2.5 _exposure	Background mortality plus health effects of additional 10 μgm^-3 ^PM_2.5 _exposure
Primary PM_2.5 _exposure scenario	The actual 2002 exposure for 2002–2112	Actual 2002 exposure for 2002, then a gradual decrease of local traffic exposure until 2025; constant thereafter	No traffic exposure between 2002–2112	Actual 2002 exposure plus 10 μgm^-3 ^for 2002–2112
Uncertainties in exposure taken into account	No	Yes	Yes	No

### Exposure-response sub-model (*β*_obs_)

An exposure-response model was built to describe the slope of the exposure-response function (*β*_obs_) of the PM_2.5 _health effects in adults and in infants. Multiple health outcomes have been detected in epidemiological studies in relation to PM_2.5_, but in this study we considered only mortality due to long-term exposure. Slopes of the exposure-response functions were estimated based on the epidemiological studies conducted in the U.S [10,25]. Also the plausibility of association was included in the model, defined as the probability that the observed exposure-response association actually represents a causal association (table 1). The plausibility of a health effect was included in the exposure-response model using author judgment based on a previous work [5]. Both plausibility and exposure-response coefficient estimates and assumptions are presented in Table 1.

There are many epidemiological studies on particulate matter and infant mortality, and recently three reviews have re-evaluated those studies [26-28]. All the reviewers concluded that there is some evidence for some association between PM levels and different mortality outcomes. The reviewers also concluded that there were many weaknesses in these published studies, e.g. variations in diagnostics between the studies [27] and lack of controlling to confounding factors (e.g. environmental tobacco smoke and other air pollutants) [27]. It should also be noted that most of the studies had used total suspended particles (TSP) or PM_10 _as a measure of the particulate air pollution, although many epidemiological studies have detected a stronger association between fine particles and health effects [e.g. [29]]. We assumed that one of the largest infant studies, the Woodruff et al. study [30], would represent best the association between traffic-related fine particles and total mortality, even though they used PM_10 _as a surrogate for particulate air pollution. The plausibility that the observed exposure-response association actually represents a causal association of the effect was assumed to be 60% (table 1).

The slope of the exposure-response function for the ages 1–110 was estimated based on epidemiological studies. There are several large epidemiological studies related to adult chronic PM_2.5 _exposure (e.g. [10,31-33]). We calculated the concentration-response coefficient by drawing values with equal probability from the result distributions reported in the Dockery et al. 1993 and Pope et al. 2002 studies (table 1) [10,25]. These two studies were selected because they had focused on overall fine particles exposure (thus, not an any specific source), they measured all the relevant health outcomes, and because we assume them to be representative for primary fine particles present the Helsinki Metropolitan Area. The equal probability was selected because we did not want to preferentially emphasize either one of these studies. The plausibility of the causal association for adult mortality was assumed separately for three mortality outcomes. We assumed that the probability for PM_2.5 _being the true cause of the effects is 70%, 90% and 10% for cardiopulmonary, lung cancer, and all other mortality, respectively. The plausibility for cancer was highest because there are known carcinogens in PM_2.5 _[34], while it is more debatable what agent is responsible for the cardiovascular effects in the air pollution mix.

### The lag sub-model (L)

Lag was defined as the time for how long a given fine particle exposure (on a yearly basis) increases the incidence of a health effect. The effect, when measured as relative risk, was assumed to be distributed evenly between exposure and the end of the lag period (see equation 1). The lag assumptions were based on the environmental tobacco smoke studies [35-38] and author judgment. For the lung cancer, cardiopulmonary, and all other non-accidental mortality, the lag was assumed to be 40, 15 and 1 years, respectively. The high lag assumptions were used to avoid any underestimation of the importance of this variable.

### Monetary valuation

The monetary value of the years-of-life-lost and the relative importance of the uncertainties related to monetary valuation were predicted to compare the relative importance of the monetary valuation on the health effect uncertainties. The monetary valuation was based on the value for a life-year lost (VOLY) estimates and discount rates that were both adopted from The Clean Air for Europe (CAFE) program.

The value of a life-year lost estimate was based on CAFE program mean and median values (52000 and 120000 euros, respectively) [39]. The CAFE values were based mainly on the NewExt study [40]. That study developed an improved methodology for undertaking a monetary valuation of mortality impacts from air pollution by using the value-of-life-year-lost approach. We adopted the values used in the CAFE program and expressed the uncertainty between the numbers with a uniform distribution (table 1).

Benefits from reduced fine particle exposure occur in the future. Discounting has been used in some fine particle health effect studies [13,14] to express the present values of these future benefits. The logic for this is that the benefits are assumed to have lower utility, if they are postponed into the distant future. Also the public seems to discount future benefits [41]. In the CAFE program 4% discount rate with 2% and 6% low and high estimates were used [39]. We adopted these numbers and expressed discount with uniform distribution ranging from 2% to 6% (table 1). Discounting was used only in monetary valuation.

### Background hazard rates (Hb)

The background hazard rates (Hb) for cardiopulmonary, lung cancer, other non-accidental, and accidental mortality were defined as the average mortality occurring over the years 1988–2002 [42] (see additional file 1: Study population.pdf for the population and mortality data). Both mortality and population data were obtained for ages 0–99. The hazard rates of the oldest age group (95–99) were used for the years 100–110. Mortality statistics had been coded using International Classification of Disease (ICD) codes version 9 (years 1988–1995) and 10 (years 1996–2002). ICD codes for cardiopulmonary, lung cancer, and accidental mortality were 390–459, 1622–1629, and 800–990, in version 9, respectively, and I00-I99, F01, and V01-Y89 in version 10. Four fifths of mortality for the first age group (0-4-year-old) was assumed to occur during the first year of life.

### Impact indicators

Life-expectancy (LE) indicator was predicted in the life-table model. The analyses were conducted for the Helsinki metropolitan area population (year 2002) of approx. 1 million inhabitants. The population age structure, including years lived before 2002, were taken into account in the life-expectancy indicator. The population was sub-divided into one-year age groups. The study population was followed until the age of 110 years.

The effect of PM exposure on mortality rate (m) ratio is typically predicted based on the following regression formula:

ln(*m*) = *α *+ *β *× *E *+ *ε*

where *α *is the background mortality coefficient, *β *is the exposure-response coefficient and E is exposure (*ε *is a nuisance parameter). Since we are interested in the effects of lag, we must assume that the observed *β *is actually a product effect resulting from a cumulative exposure that occurred before the observation. Thus,

m=exp⁡(α+∑l=0L(β×El))=Hb×exp⁡(∑l=0L(β×El))
 MathType@MTEF@5@5@+=feaafiart1ev1aaatCvAUfKttLearuWrP9MDH5MBPbIqV92AaeXatLxBI9gBaebbnrfifHhDYfgasaacH8akY=wiFfYdH8Gipec8Eeeu0xXdbba9frFj0=OqFfea0dXdd9vqai=hGuQ8kuc9pgc9s8qqaq=dirpe0xb9q8qiLsFr0=vr0=vr0dc8meaabaqaciaacaGaaeqabaqabeGadaaakeaacqWGTbqBcqGH9aqpcyGGLbqzcqGG4baEcqGGWbaCcqGGOaakiiGacqWFXoqycqGHRaWkdaaeWaqaaiabcIcaOiab=j7aIjabgEna0kabdweafnaaBaaaleaacqWGSbaBaeqaaOGaeiykaKIaeiykaKcaleaacqWGSbaBcqGH9aqpcqaIWaamaeaacqWGmbata0GaeyyeIuoakiabg2da9iabdIeaijabdkgaIjabgEna0kGbcwgaLjabcIha4jabcchaWjabcIcaOmaaqadabaGaeiikaGIae8NSdiMaey41aqRaemyrau0aaSbaaSqaaiabdYgaSbqabaaabaGaemiBaWMaeyypa0JaeGimaadabaGaemitaWeaniabggHiLdGccqGGPaqkcqGGPaqkaaa@5FF9@

where l is the time (in years) before the observation and Hb is the background hazard rate (see additional file 1: Study population.pdf for the population and mortality data).

In this article we assume that the effects of a given exposure are evenly distributed in time between the exposure and the lag L (after which no effects occur), and that in the cohort studies, the exposure was constant for at least L years. In such a scenario, we can see that:

exp⁡(β)=RRobsL
 MathType@MTEF@5@5@+=feaafiart1ev1aaatCvAUfKttLearuWrP9MDH5MBPbIqV92AaeXatLxBI9gBaebbnrfifHhDYfgasaacH8akY=wiFfYdH8Gipec8Eeeu0xXdbba9frFj0=OqFfea0dXdd9vqai=hGuQ8kuc9pgc9s8qqaq=dirpe0xb9q8qiLsFr0=vr0=vr0dc8meaabaqaciaacaGaaeqabaqabeGadaaakeaacyGGLbqzcqGG4baEcqGGWbaCcqGGOaakiiGacqWFYoGycqGGPaqkcqGH9aqpdaGcbaqaaiabdkfasjabdkfasjabd+gaVjabdkgaIjabdohaZbWcbaGaemitaWeaaaaa@3CF9@

where RRobs is the observed risk ratio per unit exposure of the exposure response function, and L is the maximum lag in years. The mortality rates (m) were estimated for each age group (ages 0–110) (*i*), year (years 2002–2112) (*y*), mortality outcome (four different outcomes) (*j*) and exposure scenarios (scenarios i-iv) (*k*). The calculated mortality rates were then used to predict the survival of the year 2002 population in the future.

The life-expectancy was predicted for the study population by summing the years lived before 2002 and years lived during the study time (years 2002–2112) and dividing the sum with the year 2002 population size. The monetary valuation was based on years-of-life-lost which differed from the LE method by considering only the years to be lived during the follow-up period and by estimating the effects of fine particle exposure using monetary indicator (euros). The differences between life-expectancy and monetary valuation impact indicators are summarized in table 3. See additional file 2 for the complete model.

**Table 3:** The main properties of two impact indicators.

	**Impact indicator**	
	**Life-expectancy**	**Monetary valuation**

Follow-up period	Years 2002–2112	
Population	Year 2002 Helsinki Metropolitan Area population	
Exposure scenarios	Yes	Yes
Years lived before 2002 taken into account	Yes	No
Monetary valuation of life years	No	Yes
Discounting	No	Yes
Sensitivity analyses	Yes	Yes

## Results

**Figure 1:**
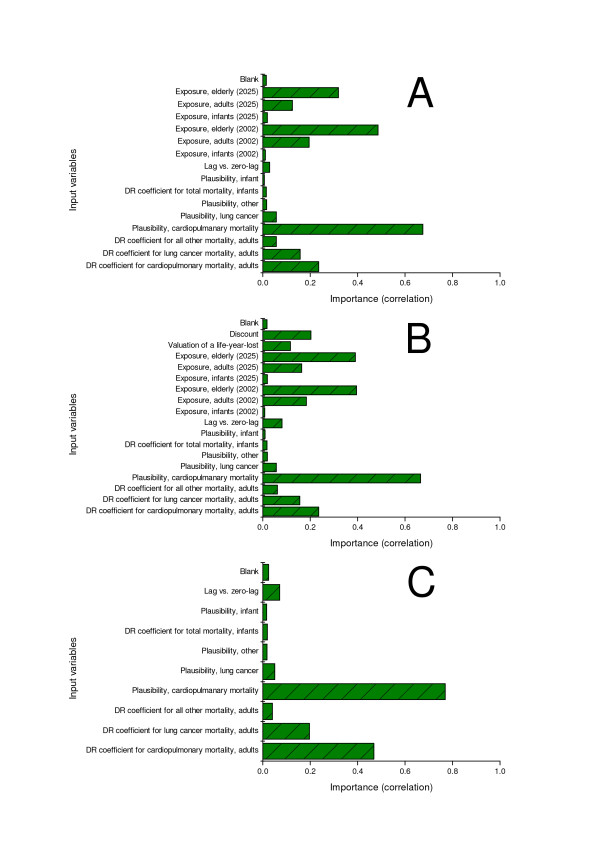
**Results from sensitivity analyses of the input variables**. The bars show how each individual input variable correlated with the model output. The high correlations indicate that the input variable has a strong impact on the model output. The results are relative, such that removal of one uncertainty will affect the sensitivity of the other uncertainties. Sensitivity analysis was done by calculating absolute rank-order correlations between the input variables and the model output. A. Impact indicator life-expectancy using the 'Current without traffic' scenario. B. Impact indicator monetary valuation using the difference between 'Current without traffic' and 'Reduced traffic emissions' scenarios. C. Impact indicator life-expectancy using the 'Additional exposure' scenario. ER = exposure-response.

Factors identified as highly important included both parameter and model uncertainties (Figure 1A–1C). The plausibility of cardiopulmonary mortality (model uncertainty) exhibited the highest importance in all scenarios. The exposure-response coefficient of cardiopulmonary mortality (parameter uncertainty) displayed high importance. Uncertainties related to lung cancer mortality and other, non-accidental, mortality had low or negligible importance. The results of the sensitivity analyses were as expected because of the high background mortality rates for cardiopulmonary mortality. The results of the sensitivity analyses were double-checked with a modified value-of-information analysis [2], in which the model result was tested against the expectation value of the model. The benefit of value of information analysis is that it is a decision analysis method that estimates the benefits of collecting additional information and expressing it in a common metric. Thus, it can be used to evaluate the importance of a particular input variable (as in this case). The value of information analysis confirmed the sensitivity analyses results (data not shown). These results highlight the importance of the cardiopulmonary mortality in comparison to the other mortality outcomes.

The plausibility and exposure-response coefficients related to infant mortality had minor importance in all sensitivity analyses (figure 1A–1C). Thus, infant mortality makes a low contribution to the population life-expectancy although the individual loss of life is considerable. However, the results would be expected to be different in developing countries due to differences in the background infant mortality rates and different cause-specific rates.

The lag vs. zero-lag input variable (model uncertainty) seemed to be of minor importance (figure 1A–C). The a priori hypothesis was that lag could be important since the Finnish population is relatively old on average, and a long lag could prevent the impact of lowering exposures from benefiting for elderly subjects. It should be noted that the lag assumptions used in this study were exceedingly long, although a few intervention studies, especially the coal ban in Dublin [31] and the Utah valley steel mill strike [43], suggest that the major part of the effect is seen within the first few years.

**Figure 2:**
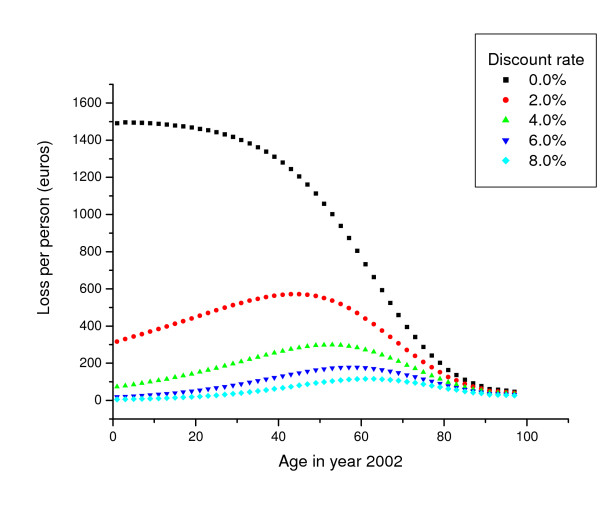
Life-years-lost costs due to fine particle exposure with different discount rates.

Discount and value of life-year-lost variables were very important in comparison to the life-expectancy uncertainties (figure 1B). The results showed how the uncertainties related to placing a monetary valuation on the effects of air pollution could have a more significant impact on the results of the model than many input variables connected to health effects evaluation. The effect of discount for the different age groups is presented in Figure 2, showing clearly how high discounts reduce the benefit to the younger age groups if compared with those over 60 years. On the other hand, the discounting did not affect the benefits to people over 80 years of age.

The continued exposure to 10 μgm^-3 ^fine particles lowered the life-expectancy of the study population by 0.41 years (mean; 90% confidence interval: 0.00 – 1.04). Previous life-table models have predicted that the continued exposure to 10 μgm^-3 ^fine particles would lowers the life-expectancy by anything between 0.42–1.5 years (table 4). The magnitude of life-expectancy loss was lower in the present study than in some of the previous life-table models. However, when the present model was run with a birth cohort of imaginary 100000 children, without plausibility estimations, and with the years 1988–1990 background hazard rates, the life-expectancy loss was 0.74 (mean, 90% CI 0.23 – 1.44) years (with 10 μgm^-3 ^PM_2.5 _exposure). Even though the model uncertainties had significant effects on model results, they were in agreement with previous models results.

**Table 4:** Comparison of the results of different life-table studies.

Study	Life-expectancy effect per 10 μgm^-3 ^(years)	Effect per predicted exposure (years)	Predicted exposure (μgm^-3^)	Relative risk estimates (mean) used in the study (per 10 μgm^-3^) (RR)
Brunekreef 1997 [7], high	1.51	1.51	10.0	1.10
Brunekreef 1997 [7], low	1.11	1.11	10.0	1.10
Nevalainen and Pekkanen 1998 [9], high	1.01	1.01	10.0	1.18^a^
Present study, high	0.74	0.74	10.0	See table 1
Mechler et al. 2002 [8]	0.64	1.36	21.1	1.06
Nevalainen and Pekkanen 1998 [9], low	0.60	0.60	10.0	1.20
Leksell and Rabl 2001 [13]	0.46	0.0006	1.0^b^	1.17
Present study, low	0.43	0.43	10.0	See table 1
Rabl 2003 [12]	0.42	0.38	15.0^c^	1.06

The results have been scaled to 10 μgm^-3 ^PM_2.5 _exposure by assuming log-linearity between increased particle exposure and loss in life-expectancy. If two or more predictions were reported, the highest and lowest numbers were selected for the table. In the present study, the low prediction is the mean of the life-table model; the high prediction is the mean of the model without plausibility assumptions, with an imaginary birthcohort and with years 1988–1990 background hazard rates. See text for details. a) Only cardiopulmonary mortality. b) Not a life-time exposure but episode of one year increased exposure. c) Estimated for PM_10_. Converted to PM2.5 by using formula 1 μg PM10 = 0.6 μg PM2.5.

The exposure of the elderly is the most important uncertainty of all the exposure variables (figure 1A–1B). This seems logical, as most of the health effects have been observed in the older age groups. The use of individual variation in exposure assessment overestimated the uncertainty of the average population exposure and could therefore lower the importance of other input variables. However, when additional analyses were conducted without exposure variability, it was noted that exposure did not significantly affect the importance of the other input variables (data not shown).

**Figure 3:**
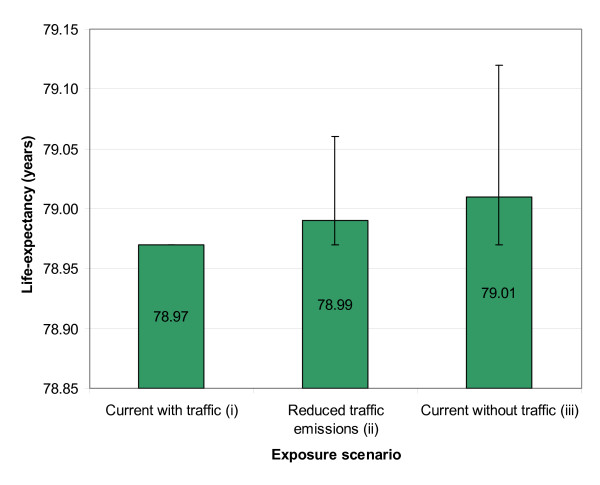
**Life-expectancy predictions for three different exposure scenarios**. Predicted life-expectancy for three different exposure scenarios (mean and 90% confidence intervals shown). The difference between scenarios 'Current without traffic' and 'Current with traffic' represents the current life-expectancy loss in Helsinki Metropolitan Area due to local traffic emitted primary fine particles emissions. The 'Reduced traffic emissions' scenario describes the effect of a plausible emission reduction scenario for 2002–2025.

The life-expectancy predictions for the three exposure scenarios are presented in Figure 3. The difference between the 'Current with traffic' and 'Current without traffic' scenarios was 0.04 years (mean, 90% CI -0.15 – 0.00), representing the life-expectancy loss in the study population due to local traffic-related primary fine particles. In absolute numbers (premature death) the local traffic was predicted to be responsible for 3.7, 27.2, and 0.2 lung cancer, cardiopulmonary and other non-accidental mortality outcomes in the year 2002, respectively. In the monetary valuation, the difference between the scenarios was 880 million euros/million inhabitants (when taking into account discount and the monetary values from the year 2002 onward). The decline in fine particles exposure by 2025, as predicted here based on the HEAT -project, improves the expected life-expectancy by 0.02 years. In the monetary valuation, the benefits were predicted to be 390 million euros/million inhabitants (from year 2002 onward). These benefits materialize in the year 2002 cohort that was followed until the year 2112. The benefits for the society are larger because the benefits to other people in the future population are not included. The local traffic-generated primary fine particles were shown to have a significant effect on health status of the study population.

## Discussion

In this study, we have made a comprehensive sensitivity analysis of a fine particle life-table model. The uncertainties relating to cardiopulmonary mortality have large effects on life-table model results, whereas the uncertainties attributable to lag and infant mortality have only minor effects. The discount and value of a life-year variables had significant impact on the results when estimating the monetary loss of the life years. The variability of exposure had major effects on the life-table model results. These results provided valuable information on the relative importance of different input variables in the life-table model and make it possible to focus on those variables and uncertainties that have the highest impact on to life-table model results and, thus, on decision making.

The sensitivity analyses showed that the life-table model result is mainly attributable to the contribution of cardiopulmonary mortality. This was expected since the background cardiopulmonary mortality is higher than lung cancer or non-accidental mortality and changes (and uncertainties) related to the concentration-response function of cardiopulmonary mortality was expected to have a major effect on the results. There are no significant differences in the magnitude of the effect between studies that have separated the mortality outcomes (present study and Nevalainen and Pekkanen study [9]) and studies that have not (all others in table 4). Cardiopulmonary mortality is declining in the developed countries, and this may affect the results from fine particle life-table models if different mortality outcomes are not separated in the analyses.

The plausibility was defined in this study as the probability that the observed exposure-response association actually represents a causal association. The separation of causal relationship and the exposure-response function have also been under examination in the recent expert elicitation study prepared by U.S. EPA [44]. The experts were asked to give their best estimate for the likelihood that there is a causal relationship between fine particles and all-cause mortality. Their answers ranged from 100% to 35%, though the majority of the experts thought that the likelihood would be 95% or more [44]. In addition to their best judgment, the expert also provided an estimate of range. Our approach differs from the EPA study, so that we separated the different mortality outcomes and used only the best estimates. As an alternative approach, we could also have tested the uncertainty around our best guess for plausibility. However, it could also be argued that plausibility is an inherently dichotomous variable and the truth is always either zero or one.

When analyzing the importance of plausibility, we used low plausibility estimates for different mortality outcomes. It could be argued that this would overestimate the current uncertainty on the plausibility of the association between fine particles, especially for lung cancer, but probably also for cardiovascular disease. With respect to lung cancer, few experts have challenged the plausibility for an association, but even for cardiovascular disease there is more and more evidence to support the existence of plausible mechanisms for an association [34,44,45]. However, the value 35%, represented in [44], shows that the plausibility is still a major issue, although the current evidence had convinces most of the experts [44]. The plausibility assumptions have been estimated in our previous study [5] and the true uncertainty based on current knowledge would probably show higher plausibility and thus have lower importance in this analysis.

In the present study, various causes of death were assumed to be statistically unrelated. The assumption means that the individuals who benefit from lowering risks (by removing the underlying cause of death) have a similar probability to survive as the individuals that were not threatened by the risk in the first place (in other words, people who benefit do not have more competing causes than the general population). It is likely that this assumption is not completely valid as such. Mackenbach et al. have investigated this question in a number of statistical studies by comparing the magnitude of competing causes in different underlying mortality outcomes from the Dutch national cause-of-death register [46-48]. In their latest analyses, they concluded that the prevalence of the competing causes is high with respect to respiratory diseases, about average with cardiovascular diseases, and low with neoplasms [48]. This implies that patients with a neoplasm have a lower risk of dying of other causes if they survive the neoplasm. If applicable to this analysis, these results could mean that the present life-table model may over-predict the import of the cardiopulmonary mortality while underestimating the import of the lung cancer.

The results from sensitivity analyses showed that both discount rate and value of life-year had a large impact on life-table model results when the results are presented using monetary indicators. The monetary valuation was also noted to be a major source of uncertainty in the CAFE uncertainty analyses, when taking into account the full chain of cost-benefit analysis [15]. The use of a monetary valuation and discount in the health risk assessment is a controversial issue. The driving force behind our analysis was to compare the uncertainties related to health effect variables with the corresponding uncertainties in monetary variables. Our intention was not to commit on discussion whether or not health effect should be valued in monetary terms. The results suggested that uncertainties related to monetary valuation are at least as important for the results as the most important health evaluation variables.

The discount rate emphasizes the importance of the health of the present population over the future generation; a principle that may be hard to accept. On the other hand, the discount rate is widely used in the financial analyses. According to economic theories, the discount rate should be consistent with all future impacts evaluated, whether health or economic. It has also been argued that the discount rate can be used in health analyses to weigh the impacts in relation to time and age. Pandey and Nathwani [49] concluded that in cost-benefit analyses, the discount rate evens out the disparity between young and old ages in life-table models. A similar result was also seen in this study when the result of the model were presented using a monetary indicator. The comparison of the benefits of different age groups showed clearly that even a low discount rate (2%) reduces the benefits to the younger age groups and relatively emphasizes effects for the middle-aged population (50 to 70 years old).

The lag has been used in some previous life-table studies. Leksell and Rabl [13] used a repair model to calculate the decline in the relative risk (repair process) after a decline in the exposure to fine particles. That model was based on the time constant, which they measured from tobacco smoking studies. Röösli et al. [50] adopted the Leksell and Rabl model, but they used air pollution intervention studies to calculate a time constant. Both models measured the association between fine particles and total non-accidental mortality. Although the authors estimated time constants based on different studies, they both concluded that it had little or no effect on life-table model results [13,50]. A similar result was seen in this study, although both the lag sub-model and the lag assumptions used were differ than those in the previous studies. The lag sub-model used in this study assumed that the adverse effect of fine particles would grow gradually while other models have used delay functions. Therefore, the results of the present study and the results from these other life-table models are not fully comparable. However, taken together, it could be concluded that lag is not an important input variable for the fine particle life-table models and therefore it could be ignored in future analyses.

Local traffic-related fine particles were shown to have a notable effect on study population. However, this was only a small part of the total exposure as the difference between PM_2.5 _exposure scenarios were about or less than 0.5 μgm^-3 ^while the total exposure in the study area is about 10 μgm^-3 ^[24]. The health effects of local traffic-related particles were also addressed only for the local population and thus the total health effects would have been larger had the dispersion of pollutants outside the study area been taken into account. Three of four exposure scenarios were based on the HEAT-project that evaluated traffic flows, air pollution emissions from traffic, resulting ambient air concentrations and exposures, and health effects in the study area. The scenarios took account of the land use policy, changes in traffic flows and emissions and the location and the time activity of the population. The comparison of the exposure scenarios showed that with the current policy, about half of the primary fine particles effects due to local traffic can be avoided within twenty years. The results show that the current actions are heading in the right direction. However, more rapid actions could be implemented, as the current transport system plan is mostly focussed on road construction and public transport subsidies and less on emission control, traffic demand, or prevention of infiltration of outdoor particles into buildings [51].

## Conclusion

The uncertainties of a life-table model for estimation of population health risks caused by local primary traffic particles were investigated in detail using a sensitivity analysis approach. The main results were: (i) uncertainties in several input variables as well as in the model structure were noted to have large effects on the model results. (ii) The uncertainty in the plausibility of the cardiopulmonary mortality, the exposure-response coefficient for cardiopulmonary mortality, and PM_2.5 _exposure in the elderly population were major sources of uncertainty, whereas (iii) uncertainty in the variables lag vs. zero-lag, exposure-response coefficient for total mortality of infants, plausibility of infant mortality effects, and other non-accidental mortality were not major sources of uncertainty. The discount and value of a life-year variables did have a significant impact on the results when comparing the results of two different exposure scenarios. The estimates used for cardiopulmonary PM coefficient, plausibility, and PM_2.5 _exposure warrant updated estimates based on the current scientific evidence. The discount rate is more of a political issue, but this analysis showed that it is important to be explicit about this issue. In contrast, complicated lag structures can be omitted without this having any major impact on the results. The fine particle emissions attributable to local traffic were shown to cause significant life-expectancy loss in the Helsinki metropolitan area population.

## Abbreviations

AJ- Author judgment

CAFE- Clean Air for Europe program

CI- Confidence interval

EPA- U.S. Environmental Protection Agency

ER- Exposure-response

EXPOLIS- Air Pollution Exposure Distributions of Adult Urban Populations in Europe -study

Hb- The background hazard rate of the study population

HEAT- Health Effects caused by Urban Air Pollution for the Transport System Plan Scenarios in Helsinki Area -study

ICD- International Classification of Disease -code

LE- Life-expectancy

NewExt- New Elements for the Assessment of External Costs from Energy Technologies -study

PM_10-_ Thoracic particulate matter (particles with aerodynamic diameter less than 10 micrometers)

PM_2.5_- Fine particulate matter (particles with aerodynamic diameter less than 2.5 micrometers)

RR- Relative risk

TSP- Total suspended particles

VOLY- Valuation of a life-year-lost

YOLL- Years-of-life-lost

YTV- Helsinki Metropolitan Area Council

## Competing interests

The author(s) declare that they have no competing interests.

## Authors' contributions

MT conceived the study, performed modeling and drafted the manuscript. JTT and OH participated in the design of the study, helped with the modeling and with the manuscript drafting. OH ran the exposure models. JR, MJJ and JP participated in the design of the study and helped with the manuscript drafting. All authors have read and approved the final manuscript.

## Supplementary Material

Additional file 1 Study population. Population, mortality and background hazard rate (Hb) statistics of the study population. Click here for file

Additional file 2 The life-table model. The model contains the full model including the code and the descriptions. ANA (Analytica™ 3.1.1. file in XML format). For a free Analytica browser, see .  Model identifier (unified resource name) is URN:NBN:fi-fe20061261. For more instructions, see . Click here for file

## References

[B1] Linkov I, Burmistrov D (2003). Model uncertainty and choices made by modelers: Lessons learned from the international atomic energy agency model intercomparisons. Risk Anal.

[B2] Morgan MG, Henrion M (1990). Uncertainty a guide to dealing with uncertainty in quantitative risk and policy analysis.

[B3] Watkiss P, Steve P, Mike H (2005). Baseline scenarios for service contract for carrying out cost-benefit analysis of air quality related issues, in particular in the clean air for Europe (CAFE) programme.

[B4] Forsberg B, Hansson HC, Johansson C, Areskoug H, Persson K, Järvholm B (2005). Comparative health impact assessment of local and regional particulate air pollutants in Scandinavia. Ambio.

[B5] Tainio M, Tuomisto JT, Hanninen O, Aarnio P, Koistinen KJ, Jantunen MJ, Pekkanen J (2005). Health effects caused by primary fine particulate matter (PM2.5) emitted from buses in the Helsinki metropolitan area, Finland. Risk Anal.

[B6] Miller BG, Hurley JF (2003). Life table methods for quantitative impact assessments in chronic mortality. J Epidemiol Community Health.

[B7] Brunekreef B (1997). Air pollution and life expectancy: is there a relation?. Occup Environ Med.

[B8] Mechler R, Amann M, Schöpp W (2002). A methodology to estimate changes in statistical life expectancy due to the control of particulate matter air pollution.

[B9] Nevalainen J, Pekkanen J (1998). The effect of particulate air pollution on life expectancy. Sci Total Environ.

[B10] Dockery DW, Pope CA, Xu X, Spengler JD, Ware JH, Fay ME, Ferris BG, Speizer et (1993). An association between air pollution and mortality in six U.S. cities. N Engl J Med.

[B11] Pope CA, Thun MJ, Namboodiri MM, Dockery DW, Evans JS, Speizer FE, Heath CW (1995). Particulate air-pollution as a predictor of mortality in a prospective-study of US adults. Am J Respir Crit Care Med.

[B12] Rabl A (2003). Interpretation of air pollution mortality: Number of deaths or years of life lost?. J Air Waste Manag Assoc.

[B13] Leksell I, Rabl A (2001). Air pollution and mortality: Quantification and valuation of years of life lost. Risk Anal.

[B14] Coyle D, Stieb D, Burnett RT, DeCivita P, Krewski D, Chen Y, Thun MJ (2003). Impact of particulate air pollution on quality-adjusted life expectancy in Canada. J Toxicol Environ Health.

[B15] Holland M, Hurley F, Hunt A, Watkiss P (2005). Methodology Paper (Volume 3) for Service Contract for carrying out cost-benefit analysis of air quality related issues, in particular in the clean air for Europe (CAFE) programme.

[B16] Kauhaniemi M, Karppinen A, Härkönen J, Kousa A, Koskentalo T, Aarnio P, Kukkonen J, Sokhi RS, Neophytou M (2007). Refinement And Statistical Evaluation Of A Modelling System For Predicting Fine Particle Concentrations In Urban Areas: 27-29 March; Limassol, Cyprur..

[B17] Hanninen OO, Lebret E, Ilacqua V, Katsouyanni K, Kunzli F, Sram RJ, Jantunen M (2004). Infiltration of ambient PM2.5 and levels of indoor generated non-ETS PM2.5 in residences of four European cities. Atmos Environ.

[B18] Jantunen M, Hänninen O, Ilacqua V, Karppinen A, Kukkonen J, Aarnio P, Sokhi RS, Millán MM, Moussiopoulos N (2005). Exposure levels to exhaust-generated and total PM2.5 in traffic: 29-31 March; Valencia, Spain..

[B19] Hanninen O, Kruize H, Lebret E, Jantunen M (2003). EXPOLIS simulation model: PM2.5 application and comparison with measurements in Helsinki. J Expo Anal Environ Epidemiol.

[B20] Kruize H, Hanninen O, Breugelmans O, Lebret E, Jantunen M (2003). Description and demonstration of the EXPOLIS simulation model: Two examples of modeling population exposure to particulate matter. J Expo Anal Environ Epidemiol.

[B21] Hanninen OO, Tuomisto JT, Jantunen MJ (2005). Characterization of model error in a simulation of fine particulate matter exposure distributions of the working age population in Helsinki, Finland. J Air Waste Manag Assoc.

[B22] Jantunen MJ, Hanninen O, Katsouyanni K, Knoppel H, Kuenzli N, Lebret E, Maroni M, Saarela K, Sram R, Zmirou D (1998). Air pollution exposure in European cities: The "EXPOLIS" study. J Expo Anal Environ Epidemiol.

[B23] Rotko T, Oglesby L, Kunzli N, Jantunen MJ (2000). Population sampling in European air pollution exposure study, EXPOLIS: comparisons between the cities and representativeness of the samples. J Expo Anal Environ Epidemiol.

[B24] Koistinen KJ, Edwards RD, Mathys P, Ruuskanen J, Kunzli N, Jantunen MJ (2004). Sources of fine particulate matter in personal exposures and residential indoor, residential outdoor and workplace microenvironments in the Helsinki phase of the EXPOLIS study. Scandinavian Journal of Work Environment & Health.

[B25] Pope CA, Burnett RT, Thun MJ, Calle EE, Krewski D, Ito K, Thurston GD (2002). Lung cancer, cardiopulmonary mortality, and long-term exposure to fine particulate air pollution. JAMA: The Journal Of The American Medical Association.

[B26] Sram RJ, Binkova BB, Dejmek J, Bobak M (2005). Ambient air pollution and pregnancy outcomes: A review of the literature. Environ Health Perspect.

[B27] Glinianaia SV, Rankin J, Bell R, Pless-Mulloli T, Howel D (2004). Does particulate air pollution contribute to infant death? A systematic review. Environ Health Perspect.

[B28] Tong SL, Colditz P (2004). Air pollution and sudden infant death syndrome: a literature review. Paediatr Perinat Epidemiol.

[B29] McDonnell WF, Nishino-Ishikawa N, Petersen FF, Chen LH, Abbey DE (2000). Relationships of mortality with the fine and coarse fractions of long-term ambient PM10 concentrations in nonsmokers. J Expo Anal Environ Epidemiol.

[B30] Woodruff TJ, Grillo J, Schoendorf KC (1997). The relationship between selected causes of postneonatal infant mortality and particulate air pollution in the United States. Environ Health Perspect.

[B31] Clancy L, Goodman P, Sinclair H, Dockery DW (2002). Effect of air-pollution control on death rates in Dublin, Ireland: an intervention study. Lancet.

[B32] Hoek G, Brunekreef B, Goldbohm S, Fischer P, van den Brandt PA (2002). Association between mortality and indicators of traffic-related air pollution in the Netherlands: a cohort study. Lancet.

[B33] Pope CA, Burnett RT, Thurston GD, Thun MJ, Calle EE, Krewski D, Godleski JJ (2004). Cardiovascular mortality and long-term exposure to particulate air pollution - Epidemiological evidence of general pathophysiological pathways of disease. Circulation.

[B34] Cohen AJ (2000). Outdoor air pollution and lung cancer. Environ Health Perspect.

[B35] Doll R, Peto R, Boreham J, Sutherland I (2004). Mortality in relation to smoking: 50 years' observations on male British doctors. Br Med J.

[B36] Dockery DW, Trichopoulos D (1997). Risk of lung cancer from environmental exposures to tobacco smoke. Cancer Causes Control.

[B37] Steenland K, Thun M, Lally C, Heath C (1996). Environmental tobacco smoke and coronary heart disease in the American Cancer Society CPS-II cohort. Circulation.

[B38] Hackshaw AK, Law MR, Wald NJ (1997). The accumulated evidence on lung cancer and environmental tobacco smoke. Br Med J.

[B39] Hurley F, Hunt A, Cowie H, Holland M, Miller B, Pye S, Watkiss P (2005). Methodology Paper (Volume 2) for Service Contract for carrying out cost-benefit analysis of air quality related issues, in particular in the clean air for Europe (CAFE) programme. Methodology for the Cost-Benefit analysis for CAFE: Volume 2: Health Impact Assessment.

[B40] IER (2004). New Elements for the Assessment of External Costs from Energy Technologies.

[B41] Cropper ML, Aydede SK, Portney PR (1994). Preferences for Life Saving Programs - How the public discounts time and age. J Risk Uncertain.

[B42] Statistics Finland (2005). Population and mortality data for year 1988-2002.

[B43] Pope CA, Schwartz J, Ransom MR (1992). Daily Mortality and PM10 Pollution in Utah Valley. Arch Environ Health.

[B44] IEC, Industrial Economics (2006). Expanded Expert Judgment Assessment of the Concentration-Response Relationship Between PM2.5 Exposure and Mortality.

[B45] Brook RD, Franklin B, Cascio W, Hong Y, Howard G, Lipsett M, Luepker R, Mittleman M, Samet J, Smith SC, Tager I (2004). Air pollution and cardiovascular disease: a statement for healthcare professionals from the Expert Panel on Population and Prevention Science of the American Heart Association. Circulation.

[B46] Mackenbach JP, Kunst AE, Lautenbach H, Bijlsma F, Oei YB (1995). Competing causes of death - An analysis using multiple-cause-of-death data from the Netherlands. Am J Epidemiol.

[B47] Mackenbach JP, Kunst AE, Lautenbach H, Oei YB, Bijlsma F (1997). Competing causes of death: A death certificate study. J Clin Epidemiol.

[B48] Mackenbach JP, Kunst AE, Lautenbach H, Oei YB, Bijlsma F (1999). Gains in life expectancy after elimination of major causes of death: revised estimates taking into account the effect of competing causes. J Epidemiol Community Health.

[B49] Pandey MD, Nathwani JS (2003). Canada wide standard for particulate matter and ozone: Cost-benefit analysis using a life quality index. Risk Anal.

[B50] Röösli M, Künzli N, Braun-Fahrländer C, Egger M (2005). Years of life lost attributable to air pollution in Switzerland: dynamic exposure-response model. Int J Epidemiol.

[B51] YTV (2002). Helsinki Metropolitan Area transport system plan PLJ 2002. The Helsinki Metropolitan Area Publication Series A.

