# Supplementary material for: Parameter and model uncertainty in a life-table model for fine particles (PM2.5): a statistical modeling study
Source: Environ Health. 2007 Aug 23;6:24. doi: 10.1186/1476-069X-6-24 (PMC2000460; doi:10.1186/1476-069X-6-24)
Supplement: Additional file 1 — Study population. Population, mortality and background hazard rate (Hb) statistics of the study population. [file 1476-069X-6-24-S1.pdf]

Additional file 1: Population, mortality and background hazard rate (Hb) statistics of the study population. Article: Tainio et al. Parameter and model uncertainty in a life-table model for fine particles (PM2.5): a statistical modelling study (2007).

Age Group	Population in year 2002 (no.)	Total mortality	Lung cancer mortality		Cardiopulmonary mortality		Other non-accidental mortality		Accidental mortality	
		Hb	no.	Hb	no.	Hb	no.	Hb	no.	Hb
**0**	11 560	4.2E-03	0	0.0E+00	0	4.2E-05	45	3.9E-03	3	2.4E-04
**1-4**	43 960	2.7E-04	0	0.0E+00	0	2.7E-06	11	2.6E-04	1	1.6E-05
**5-9**	56 530	1.1E-04	0	0.0E+00	0	7.1E-06	3	5.4E-05	3	4.7E-05
**10-14**	55 640	1.2E-04	0	0.0E+00	1	1.1E-05	4	7.2E-05	2	3.4E-05
**15-19**	51 800	4.4E-04	0	2.6E-06	0	9.0E-06	5	1.1E-04	17	3.3E-04
**20-24**	72 710	6.5E-04	0	0.0E+00	2	2.8E-05	11	1.6E-04	34	4.6E-04
**25-29**	83 120	7.2E-04	0	1.6E-06	4	5.1E-05	15	1.8E-04	41	5.0E-04
**30-34**	77 350	1.1E-03	0	4.3E-06	8	1.1E-04	27	3.4E-04	52	6.7E-04
**35-39**	82 360	1.5E-03	1	1.2E-05	18	2.2E-04	47	5.7E-04	61	7.4E-04
**40-44**	72 820	2.5E-03	4	5.4E-05	35	4.7E-04	75	1.0E-03	70	9.5E-04
**45-49**	68 660	3.7E-03	7	1.0E-04	59	8.6E-04	112	1.6E-03	78	1.1E-03
**50-54**	71 140	4.4E-03	17	2.4E-04	86	1.2E-03	144	2.0E-03	66	9.2E-04
**55-59**	67 580	5.2E-03	25	3.8E-04	113	1.7E-03	160	2.4E-03	55	8.1E-04
**60-64**	44 310	1.0E-02	39	8.8E-04	176	4.0E-03	200	4.5E-03	45	1.0E-03
**65-69**	33 600	1.8E-02	50	1.5E-03	255	7.6E-03	261	7.8E-03	35	1.0E-03
**70-74**	29 160	2.7E-02	57	2.0E-03	361	1.2E-02	343	1.2E-02	32	1.1E-03
**75-79**	22 150	4.5E-02	51	2.3E-03	501	2.3E-02	419	1.9E-02	30	1.4E-03
**80-84**	14 850	7.8E-02	33	2.2E-03	616	4.1E-02	472	3.2E-02	35	2.4E-03
**85-89**	8 435	1.2E-01	13	1.6E-03	548	6.5E-02	420	5.0E-02	30	3.6E-03
**90-94**	3 376	1.6E-01	4	1.1E-03	282	8.3E-02	227	6.7E-02	16	4.9E-03
**95-99**	665	2.3E-01	1	8.0E-04	72	1.1E-01	73	1.1E-01	4	6.7E-03
**100-104**	0	0.0E+00	0	0.0E+00	5	0.0E+00	5	0.0E+00	1	0.0E+00
**105-110**	0	0.0E+00	0	0.0E+00	0	0.0E+00	0	0.0E+00	0	0.0E+00
**Sum**	**971 776**		**304**	**-**	**3142**	**-**	**3078**	**-**	**709**	**-**
